# Reflections on NICE Headache Guidelines

**DOI:** 10.1186/1129-2377-14-S1-O4

**Published:** 2013-02-21

**Authors:** Kay Kennis

**Affiliations:** 1 Moorside Surgery, 370 Dudley Hill Road, Bradford, UK

## 

The presentation illustrates the process for the development of the recent NICE Headache Guidelines and the importance of careful topic selection. The methodology, timescale, and the role of the Expert Group including patient representatives are described. The Guidelines are intended for the non-specialist in Primary Care where most patients present and can be safely diagnosed and managed.

The Guidelines provide support with the diagnosis of primary headache including the value of neuroimaging which evidence suggests should not be used only for reassurance. The importance of excluding secondary causes, in particular medication overuse which is found among migraine sufferers is emphasised. Specific advice is given for management of the three most common types of primary headache; migraine, tension headache and cluster headache. Changes are recommended to current practice including prescribing combination therapy in acute migraine and indications for prophylactic topiramate. The management of female migraine sufferers of child baring potential also receives attention and caution is advised in the use of combined hormonal contraception for patients with aura due to increased risk of stroke. There are also recommendations for perimenstrual prophylaxis with frovatriptan or zolmitriptan.

Improved recognition of common headache disorders and better targeting of treatments should reduce the burden of headache without requiring substantial extra resource. Where more specialised advice is required the Guideline recommends referral to a neurologist or GP with special interest in headache.

